# Kinetics and thermodynamics of the protein-ligand interactions in the riboflavin kinase activity of the FAD synthetase from *Corynebacterium ammoniagenes*

**DOI:** 10.1038/s41598-017-07875-5

**Published:** 2017-08-04

**Authors:** María Sebastián, Ana Serrano, Adrián Velázquez-Campoy, Milagros Medina

**Affiliations:** 1 Department of Biochemistry and Molecular and Cellular Biology, Faculty of Sciences, and Institute of Biocomputation and Physics of Complex Systems (Joint Units: BIFI-IQFR and GBsC-CSIC), University of Zaragoza, Zaragoza, Spain; 2 ARAID Foundation, Diputación General de Aragón, Zaragoza, Spain; 3 Aragon Institute for Health Research (IIS Aragon), Zaragoza, Spain; 4 Centro de Investigaciones Biológicas, CSIC, Ramiro de Maeztu 9, Madrid, Spain

## Abstract

Enzymes known as bifunctional and bimodular prokaryotic type-I FAD synthetase (FADS) exhibit ATP:riboflavin kinase (RFK) and FMN:ATP adenylyltransferase (FMNAT) activities in their C-terminal and N-terminal modules, respectively, and produce flavin mononucleotide (FMN) and flavin adenine dinucleotide (FAD). These act as cofactors of a plethora of flavoproteins in all organisms. Therefore, regulation of their production maintains the cellular flavoproteome homeostasis. Here, we focus on regulation of the FMN synthesis in *Corynebacterium ammoniagenes* (*Ca*) by the inhibition of its RFK activity by substrates and products of the reaction. We use a truncated *Ca*FADS variant consisting in the isolated C-terminal RFK module, whose RFK activity is similar to that of the full-length enzyme. Inhibition of the RFK activity by the RF substrate is independent of the FMNAT module, and FMN production, in addition to being inhibited by an excess of RF, is also inhibited by both of the reaction products. Pre-steady-state kinetic and thermodynamic studies reveal key aspects to the substrates induced fit to produce the catalytically competent complex. Among them, the role of Mg^2+^ in the concerted allocation of substrates for catalysis and the ensemble of non-competent complexes that contribute to the regulated inhibition of the RFK activity are particularly relevant.

## Introduction

Flavin mononucleotide (FMN) and flavin adenine dinucleotide (FAD) derive from riboflavin (RF, vitamin B2) and act as cofactors of flavoproteins. These proteins play central roles in a plethora of metabolic processes that are relevant in energetic metabolism, synthesis and degradation of proteins, neuronal development and apoptosis^1^. FMN or FAD deficiency leads to accumulation of apoflavoproteins that are unable to perform their functions, with the death of the organism as final consequence^1–3^. In this sense, FMN and FAD biosynthesis appear to be metabolic processes with capital relevance to different biological perspectives. On one hand, the overall process must be highly regulated since the excess or lack of these flavins entails the alteration of the cellular flavoproteome homeostasis^4–6^. In addition, flavin synthesis is an attractive exploitable target since development of specific inhibitors that interfere with flavin production in pathogenic organisms might compromise their life cycle without affecting the host^3^.

Biosynthesis of FMN and FAD from RF involves two reactions: RF is first phosphorylated to FMN in an ATP-Mg^2+^-dependent reaction carried out by an ATP:riboflavin kinase (RFK), and then an FMN:ATP adenylyltransferase (FMNAT) transfers the adenylyl group from a second ATP molecule to FMN to yield FAD^7, 8^. In eukaryotes, these reactions are preferentially performed by two independent monofunctional enzymes^9, 10^, but in most prokaryotes, the two reactions are sequentially catalyzed by a bifunctional enzyme known as prokaryotic type I FAD synthetase (FADS)^11–13^. These bifunctional proteins are organized in two nearly independent modules, with each one catalyzing one of the two activities. The C-terminal module catalyzes the RFK activity, while the N-terminal module carries out the FMNAT reaction; thus, they are also known as the RFK module and the FMNAT module, respectively. The FADS from *Corynebacterium ammoniagenes* (*Ca*FADS) is hitherto the best characterized member of this family from both functional and structural points of view^12, 14–19^. One key feature of *Ca*FADS is the strong inhibition that is observed in its RFK activity when the RF substrate concentration is increased^20, 21^. The RFK module is formed by residues 187–338 and shows a globular shape with a β-barrel formed by six antiparallel strands, a terminal α-helix that is perpendicular to the barrel and seven loops connecting them^15^. Despite the strong sequential and structural homology with the RFK module of other bacterial enzymes and with the corresponding monofunctional mammalian enzymes, some dissimilar characteristics are expected among the few studied members of this family^6, 12, 13, 22, 23^. Among them, we should remark the lack of substrate inhibition reported for family members different from *C*. *ammoniagenes*; another difference lies in the dissimilar conformational reorganizations that have been predicted to occur during catalysis^14, 18, 24^. Both facts are of great biological importance since many metabolic pathways are regulated through selective inhibition or conformational changes in the implicated enzymes^25–28^. In addition, the understanding of these processes is of general interest for the discovery of potential drugs that might act as inhibitors of prokaryotic FADSs.

In the present study, we focus on these two particular facts while characterizing the isolated RFK module of *Ca*FADS (Δ(1–182)*Ca*FADS). This truncated form of the enzyme has shown to perform the RFK activity with ligand binding profiles and strong substrate inhibition that are similar to those observed in the full-length bifunctional enzyme^18^. Moreover, considering the intrinsic difficulties that are inherent to some of the techniques used here, the isolated RFK module is a simpler model for the thorough study of the regulation of RFK activity in *Ca*FADS. Herein, we present a steady-state study that shows the different levels and potency at which substrates and products of the RFK module inhibit its activity. In addition, the use of fast kinetics methods sheds light on the sequential conformational changes elicited in the protein by substrates and products during binding and catalysis. Finally, isothermal titration calorimetry experiments outline the intricate ensemble of protein complexes to identify the most favorable pathways and to predict cooperativity between ligands. Collectively, these results are discussed in the framework of the crystal structures of the RFK module of *Ca*FADS, both free and in complex with its products^14, 18^, to evaluate the conformational changes that are produced during ligand binding and RFK catalysis.

## Results

### The RFK activity is inhibited by the RF substrate as well as the products of the reaction

**Figure 1:**
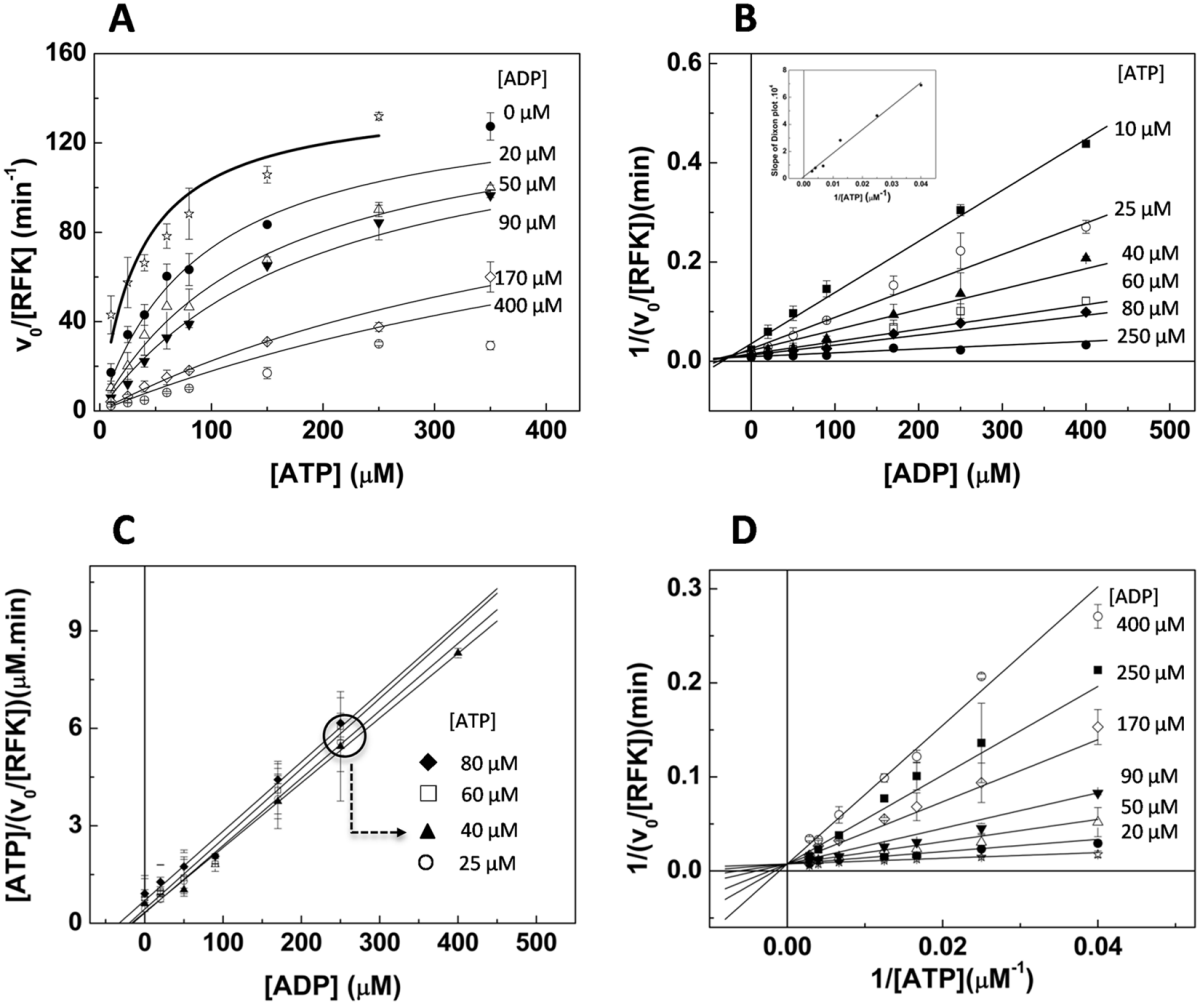
Inhibitory effects produced by the ATP substrate and the ADP product in the steady-state RFK activity of the RFK module of *Ca*FADS. (**A**) Michaelis-Menten plots as a function of the ATP substrate concentration at different concentrations of the ADP product, and the corresponding (**B**) Dixon plot with the representation of the slopes *vs* 1/[ATP] in the inset, (**C**) the corresponding Cornish-Bowden plot and (**D**) the Lineweaver-Burk representation. In (**A**, ** C** and **D**) lines correspond to global fits to the equation for competitive inhibition. Reaction rates were obtained in 20 mM PIPES and 0.8 mM MgCl_2_, pH 7.0, at 25 °C, with 5 μM RF (concentration at which the enzyme shows 80% of its maximal apparent activity at saturating levels of ATP), while the concentrations of the ATP substrate (10–350 μM) and the ADP product (0–400 μM) were varied.

**Figure 2:**
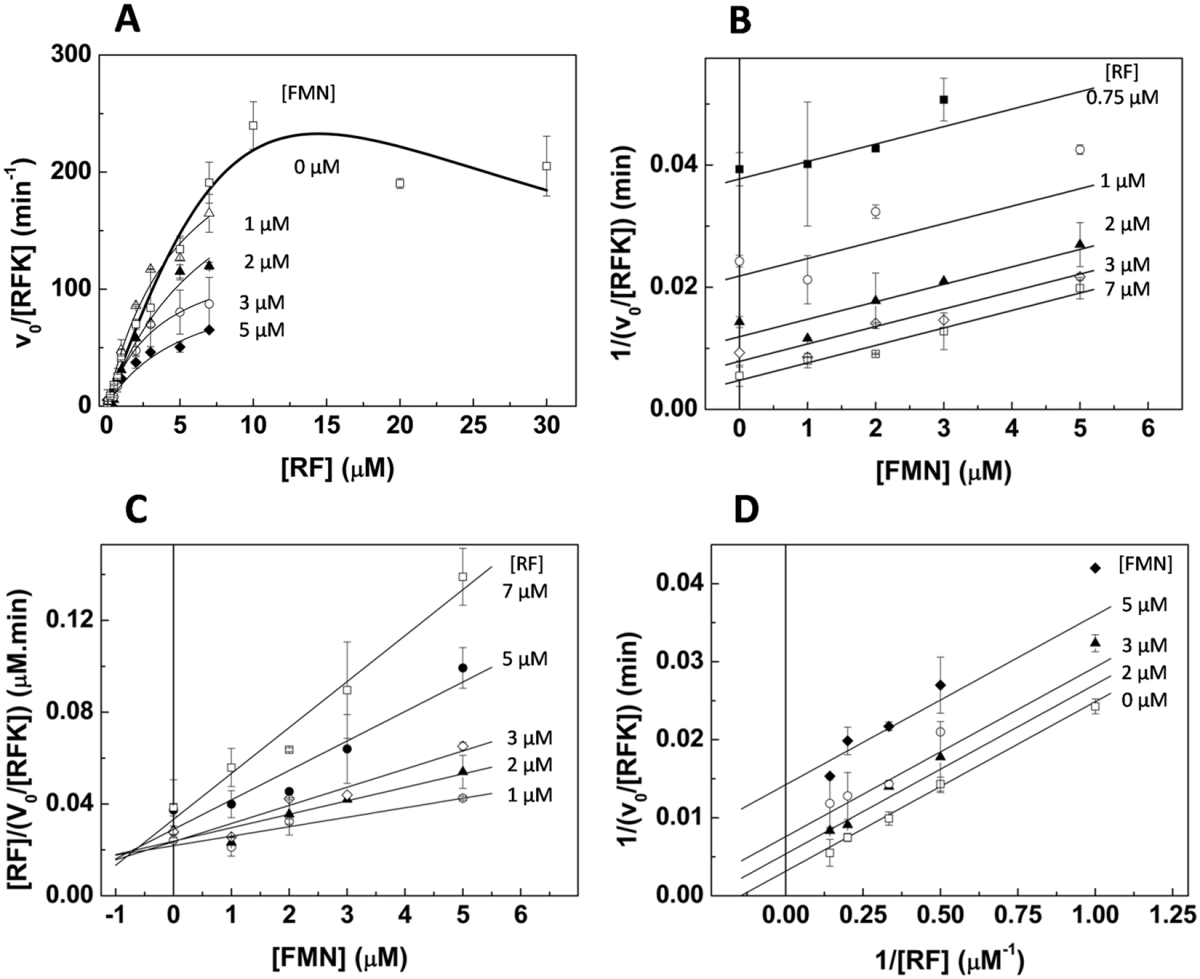
Inhibitory effects produced by the RF substrate and the FMN product in the steady-state RFK activity of the RFK module of *Ca*FADS. (**A**) Michaelis-Menten plots as a function of the RF substrate concentration at different concentrations of the FMN product (high fluorescence yields in mixtures that simultaneously contained RF and FMN prevented accurate determination of data over 7 µM RF) and the corresponding (**B**) Dixon plot, (**C**) Cornish-Bowden plot and (**D**) Lineweaver-Burk representation. In (**A**, **C** and **D**) lines correspond to global fits to the equation for uncompetitive inhibition. Reaction rates were obtained in 20 mM PIPES and 0.8 mM MgCl_2_, pH 7.0, at 25 °C and a saturating level of ATP (350 μM ATP), while the concentrations of the RF substrate (0–30 μM) and the FMN product (0–7 μM) were varied.

The independently expressed RFK module of *Ca*FADS retains the ability to catalyze the phosphorylation of RF and conserves the structural determinants that are responsible for strong inhibition by its RF substrate (bold lines in Figs 1A and 2A)^18^. Fitting of the observed steady-state rates to either the Michaelis-Menten equation (Fig. 1, bold line in panel A) or the equation describing dead-end inhibition by substrate excess (Eqn. 1) (Fig. 2, bold line in panel A) allowed the determination of the apparent values for the constants that characterize the process, with ${K}_{m}^{ATP}$, ${K}_{m}^{RF}$, ${K}_{i}^{RF}$ and *k*_cat_ values of 60 ± 11 µM, 10 ± 2 µM, 1.9 ± 0.2 µM and up to ∼440 min^−1^, respectively (Table 1).

**Table 1:** Steady-state kinetic parameters describing the RFK activity of the RFK module and the inhibition by its different substrates and products.

Kinetic parameters as determined in the absence of products of the reaction						
[ADP] (μM)	*k*_*cat*_^a^ (min^−1^)	${{\boldsymbol{K}}}_{{\boldsymbol{m}}}^{{\boldsymbol{ATP}}}$ ^a^ (μM)	[FMN] (μM)	*k*_*cat*_^b^ (min^−1^)	${{\boldsymbol{K}}}_{{\boldsymbol{m}}}^{{\boldsymbol{RF}}}$ ^b^ (μM)	${{\boldsymbol{K}}}_{{\boldsymbol{i}}}^{{\boldsymbol{RF}}}$ ^b^ (μM)
0	160 ± 10	60 ± 11	0	440 ± 50	10 ± 2	1.9 ± 0.2
**Kinetic parameters as determined at different concentrations of reaction products**						
20	160 ± 10	110 ± 20	1	280 ± 40	5.0 ± 1.1	
50	140 ± 10	140 ± 20	2	270 ± 50	7.7 ± 1.4	
90	170 ± 10	260 ± 20	3	140 ± 30	3.6 ± 0.9	
170	170 ± 60	700 ± 270	5	120 ± 40	5.5 ± 2.1	
**Kinetic parameters determined by globally fitting the Lineweaver-Burk equation**						
${{\boldsymbol{K}}}_{{\boldsymbol{i}}}^{{\boldsymbol{ADP}}}$ ^**a**^ **(μM)**	***k***_***cat***_^**a**^ **(min** ^**−1**^ **)**	${{\boldsymbol{K}}}_{{\boldsymbol{m}}}^{{\boldsymbol{ATP}}}$ ^**a**^ **(μM)**	${{\boldsymbol{K}}}_{{\boldsymbol{i}}}^{{\boldsymbol{FNM}}}$ ^**b**^ **(μM)**	***k***_***cat***_^**b**^ **(min** ^**−1**^ **)**	${{\boldsymbol{K}}}_{{\boldsymbol{m}}}^{{\boldsymbol{RF}}}$ ^**b**^ **(μM)**	
17.0 ± 3.3	130 ± 30	40 ± 12	1.4 ± 0.2	320 ± 30	6.9 ± 0.4	

Data obtained at 25 °C in 20 mM PIPES and 0.8 mM MgCl_2_, pH 7.0. Inhibition by the RF substrate prevented the determination of true parameters, and the data that is presented correspond to apparent constants.

^a^Parameters estimated using an RF concentration at which activity was ∼80% of maximal(~5 µM).

^b^Determined at saturating ATP concentrations.

To evaluate whether any of the products of the RFK reaction also produce inhibition, we analyzed the evolution of the steady-state rates at increasing concentrations of either ADP or FMN (Figs 1 and 2). The fitting of the rates, obtained as a function of the ATP concentration while varying the ADP concentration, to the Michaelis-Menten model shows that while ${K}_{m}^{ATP}$ values increase with the ADP concentration, *k*_*cat*_ values remain constant (Fig. 1A and Table 1). Dixon and Cornish-Bowden plots for these data were then used to identify the type of inhibition produced by ADP. The Dixon plot shows lines intersecting at negative values of the x-axis, while in the Cornish-Bowden representation, the lines are parallel (Fig. 1B and C). These plots reveal that the ADP product acts as a competitive inhibitor. Plotting the slopes from the Dixon plot *vs* 1/[ATP] confirms a pure competitive inhibition (Fig. 1B, inset). A global fit of the data in the Lineweaver-Burk representation to the equation describing competitive inhibition (Eqn. 2), yields *k*_*cat*_, ${K}_{m}^{ATP}\,$and ${K}_{i}^{ADP}$ values of 130 ± 30 min^−1^, 40 ± 20 μM and 17 ± 6 μM, respectively (Fig. 1A and D and Table 1). In this case, the high affinity for the ADP product inhibitor considerably increases the estimated error for ${K}_{m}^{ATP}$.

The fits of the observed rates *vs* RF to the Michaelis-Menten equation, in experiments carried out at saturating ATP and increasing FMN concentrations, show that *k*_*cat*_ values decrease and ${K}_{m}^{RF}$ values do not significantly change (Table 1 and Fig. 2A). This result indicates that the FMN product also behaves as an inhibitor of RFK activity. In this case, the Dixon plot displays parallel lines (Fig. 2B), while lines intersect at negative values of the x-axis in the Cornish-Bowden graphic (Fig. 2B and C). These plots are typical of uncompetitive inhibitors. A global fit of the Lineweaver-Burk plots to the uncompetitive inhibition equation (Eqn. 3) yields 320 ± 40 min^−1^ for *k*_*cat*_, 6.9 ± 0.4 μM for ${K}_{m}^{RF}$ and 1.4 ± 0.2 μM for ${K}_{i}^{FMN}$ (Fig. 2D and Table 1).

These data indicate that the RFK activity is inhibited by the RF substrate, as well as by both products of the reaction, with ADP acting as a competitive inhibitor and FMN as an uncompetitive inhibitor.

### Changes in flavin fluorescence upon binding allow the association and dissociation kinetics of flavin ligands to the RFK module to be studied

We used stopped-flow spectrophotometry to kinetically differentiate some of the steps that were implicated in the RFK reaction under pre-steady-state situations. These processes can be identified by changes in the fluorescence of the flavin isoalloxazine ring, a property that is highly dependent on the ring’s environment^29^. Since RF and FMN share the same fluorescence spectrum and yields^30^, direct transformation of RF into FMN is not expected to be observed by this method. However, we should be able to detect processes that induce changes in the electronic environment of the flavin isoalloxazine ring, including flavin binding, flavin dissociation and even any conformational change around the isoalloxazine that occurs during catalysis. When we mix RF or FMN (herein FLV will be used to denote either one of them) ligands with the protein in the stopped-flow equipment and follow the evolution of flavin fluorescence, we only detect slow linear fluorescence decays, whose slopes linearly decrease with the FLV concentration and are consistent with the photobleaching range (rates of 4.5 · 10^−3^ ± 1.2 · 10^−4^ min^−1^ for RF and 4.1 · 10^−3^ ± 2.4 · 10^−4^ min^−1^ for FMN) (see methods and Fig. SP1). These results reveal that the RFK module is not able to directly bind the FLV ligand and/or to internalize its isoalloxazine ring into the active site in a competent, enclosed conformation.

**Figure 3:**
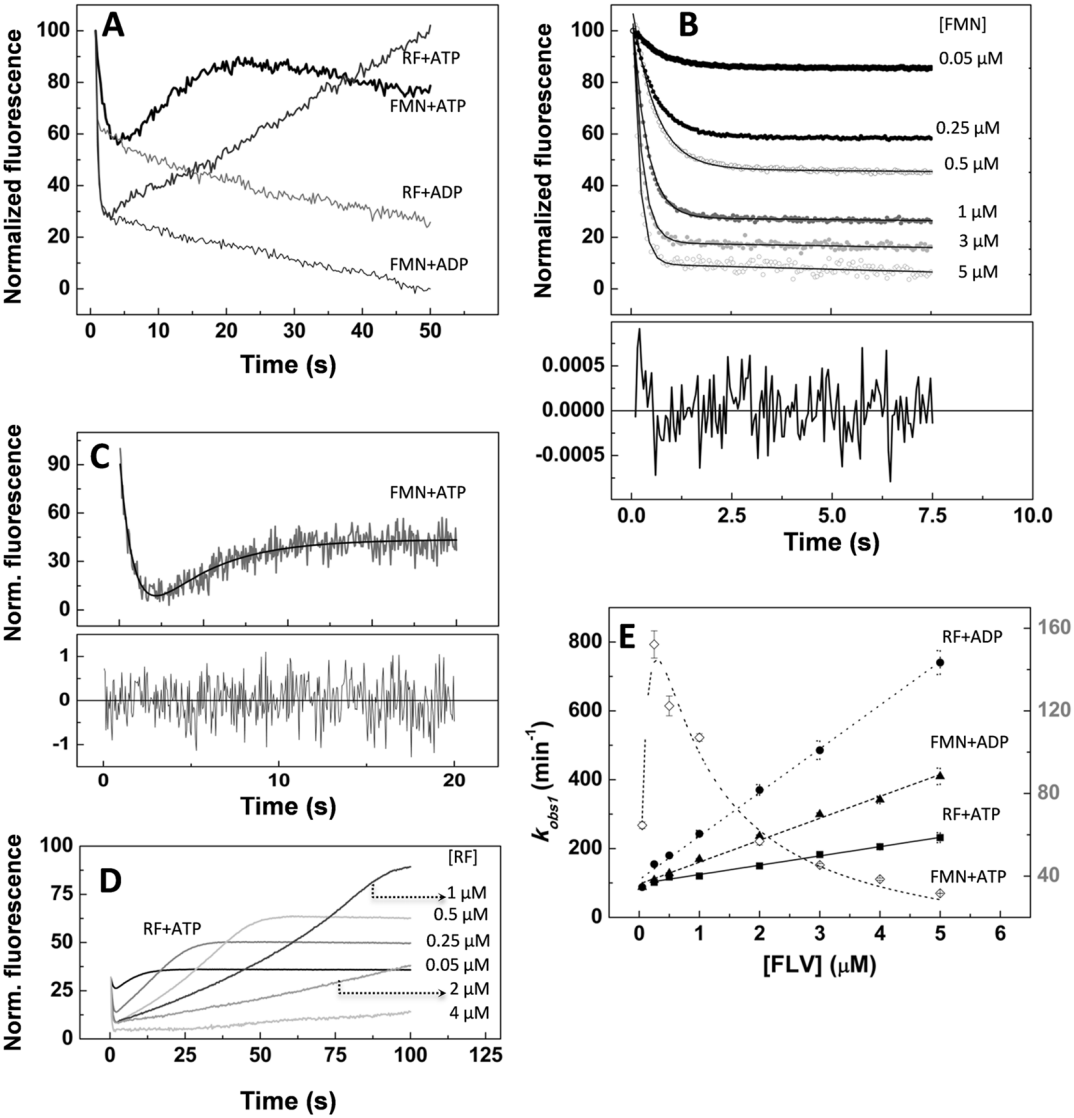
Pre-steady-state kinetic analysis of the binding of flavins to the RFK module of *Ca*FADS. (**A**) Kinetic traces of the evolution of flavin fluorescence upon mixing the RFK module (0.2 μM) with RF (1 μM) at saturating levels of ATP (250 μM) and ADP (250 μM) as well as samples of the RFK module with FMN (1 μM) at saturating levels of ATP (250 μM) and ADP (250 μM). (**B**) Evolution of kinetic traces of FMN interacting with the RFK module in the presence of 250 μM ADP at various FMN concentrations. Traces are fit to a mono-exponential decay with a steady-state component. Residuals of fitting the 1 μM FMN data are shown at the bottom. (**C**) Dissection of the exponential processes for kinetic traces that were observed when the RFK module (0.2 μM) containing a saturating level of ATP (250 μM) was mixed with FMN (1 μM). The panel shows the fit to a bi-exponential process, and the fitting residual is shown at the bottom. (**D**) Traces obtained when mixing the RFK module (0.2 μM) containing a saturating level of ATP (250 μM) with different RF concentrations. (**E**) Evolution of *k*_*obs1*_ as a function of the flavin concentration for the mixing of the RFK module with RF + ADP (●), FMN + ADP (▲), RF + ATP (RFK reaction, ■) and (**C**) FMN + ATP (◊). For those data showing linear dependences, the slopes are related to the *k*_*on*_ for the binding, and the independent term is associated with the *k*_*off*_. All the experiments were carried out in the stopped-flow equipment at 25 °C in 20 mM PIPES and 0.8 mM MgCl_2_, pH 7.0. All concentrations that are indicated are final concentrations in the mixing cell.

Kinetic traces were then recorded when the RFK module was mixed with different combinations of flavin and adenine nucleotide ligands. These traces show kinetic processes taking place faster than those related to flavin photobleaching. The number of processes that were detected depended on the ligands that were present in the mixture (Fig. 3A–D). All the analyzed mixtures showed a fluorescence decay in the 5-s time frame that fits to a single exponential process, allowing the determination of the corresponding *k*_*obs1*_ values (Fig. 3A–D, see a highlighted FMN example in Fig. 3B). This feature was not observed when flavins are independently mixed with adenine nucleotide ligands (ANP herein denotes either ADP or ATP) in absence of the RFK module (not shown) or with the RFK module in the absence of ANP ligands (Fig. SP1A). Therefore, this process can be identified as the ANP promoting changes in the polar environment of the isoalloxazine ring and must be related to flavin binding and/or to its internalization within the protein matrix. In general, *k*_*obs1*_ values are linearly dependent on the FLV concentration (Fig. 3E). The fit of *k*_*obs1*_ values to Eqn. 6 yields the flavin association rates, *k*_*on*_, as the slope and the corresponding dissociation rates, *k*_*off*_, as the independent term. These values are reported in Table 2 and show how, at saturating concentrations of the ADP product, the RF binding is two times faster than the FMN binding. In contrast, the binding of the substrates of the RFK reaction, RF and ATP, is the slowest process. Thus, apparently, the products of the reaction bind the RFK module faster than the substrates do, and as consequence, substrate binding appears slightly weaker (Table 2). Noticeably, *k*_*obs1*_ values for non-productive mixtures containing the FMN product and the ATP substrate are considerably slower than the others and show a biphasic dependence on the FMN concentration that prevents the determination of *k*_*on*_ and *k*_*off*_ (Fig. 3D). This behavior indicates that an excess of FMN in the presence of ATP hinders its own binding and/or a conformational change in the flavin environment.

**Table 2:** Pre-steady-state kinetic parameters for the binding and dissociation of flavins to the RFK module in the presence of adenine nucleotides.

**Ligand combination**	***k***_***on***_ **(min** ^**−1**^ **μM** ^**−1**^ **)**	***k***_***off***_ **(min** ^**−1**^ **)**	***K***_***d***_ **(μM)**	**Δ** ***G*** **(kcal/mol)**
FMN-ADP	64 ± 2	97 ± 5	1.5 ± 0.1	−7.9 ± 0.5
RF-ADP	130 ± 3	110 ± 8	0.9 ± 0.1	−8.2 ± 0.1
RF-ATP	27 ± 1	98 ± 3	3.6 ± 0.2	−7.4 ± 0.3
FMN-ATP	NA	NA	NA	

Experiments were carried out in 20 mM PIPES and 0.8 mM MgCl_2_, pH 7.0. NA, not applicable.

In experiments performed with ADP, after the initial decay, here related to flavin recognition in the presence of the ANP, only slow decays are observed, which are consistent with flavin photobleaching and therefore assumed to not be part of the reaction. However, experiments carried out with mixtures of FLV and ATP show an exponential increase in fluorescence, characterized by *k*_*obs2*_ (Fig. 3A,C and D), before the photobleaching decay starts. These observations suggest that after the initial interaction of ligands with the protein, a subsequent ATP-dependent conformational rearrangement is produced in the flavin environment. Such a rearrangement brings back the isoalloxazine ring to a solvent accessible environment. Interestingly, both the amplitude and *k*_*obs2*_ values increase with the flavin concentration at concentrations below 0.25–0.5 µM but considerably decrease at higher flavin concentrations (Figs 3C and SP2). This biphasic profile agrees with the behavior of excess FLV ligands inhibiting the catalytic process (see Fig. 2A). These data also indicate that this second process is related to the release of the isoalloxazine ring to the solvent, which, according to the *k*_*obs2*_ values, appears to be the limiting step in the catalytic reaction.

Altogether, and despite differences in experimental settings, these data show that the fast kinetics rates, *k*_obs1_ and *k*_obs2_, for processes that are compatible with RFK catalysis (containing RF, ATP, and Mg^2+^) are close to the *k*_cat_ values (Fig. 3D–E and Table 1). This similarity indicates that the pre-steady-state kinetics that are described here are relevant for catalysis and the final return of the flavin to the solvent is the limiting step of the reaction.

### The thermodynamic diagram for the RFK-ligand interactions

We performed ITC experiments to verify that the observed pre-steady-state kinetic processes were significant in reaching the thermodynamic equilibrium. Titrations with the different ligands (substrates and products) of the isolated RFK module or of its mixtures with either FLV or ANP ligands provide values of the thermodynamic parameters (Gibbs free energy (Δ*G*), enthalpy (Δ*H*) and entropy (−TΔ*S*), as well as the corresponding *K*_*d*_) for each binary and ternary interaction. The corresponding numeric data are summarized in Table SP1, while thermodynamic dissections and some examples of the titrations are displayed in Figs SP3 and SP4. Titrations were performed in 0.8 mM MgCl_2_ (conditions showing the highest efficiency for the RFK activity)^11^ (Fig. SP3A and upper panel Table SP1), as well as in the absence of MgCl_2_ to avoid catalysis when both substrates, RF and ATP, are present (the catalytic reaction heat would mask the binding enthalpy) (Fig. SP3B and lower panel in Table SP1).

Thermograms for the titration of the RFK module with the individual ligands indicate that: (i) FMN does not interact or interacts with undetectable Δ*Cp* (no interaction at all was detected when titrating at the different temperatures); (ii) RF, ATP and ADP bind with appreciable enthalpic changes; and (iii) the presence of the Mg^2+^ cation modulates binding parameters (Table SP1). When mixtures containing the RFK module and ANP (ATP or ADP) are titrated with FLV (RF or FMN), or *vice versa*, Mg^2+^ favors formation of ternary complexes, which, as the more negative free binding energies show, are more stable in the presence of the cation. This positive effect of the cation on the enzyme affinity for ligands in ternary complexes is, in general, a consequence of the less unfavorable entropic contribution to the binding (Table SP1 and Fig. SP3). Therefore, Mg^2+^ contributes to the overall conformation of the system in binary and particularly, ternary, interactions.

**Figure 4:**
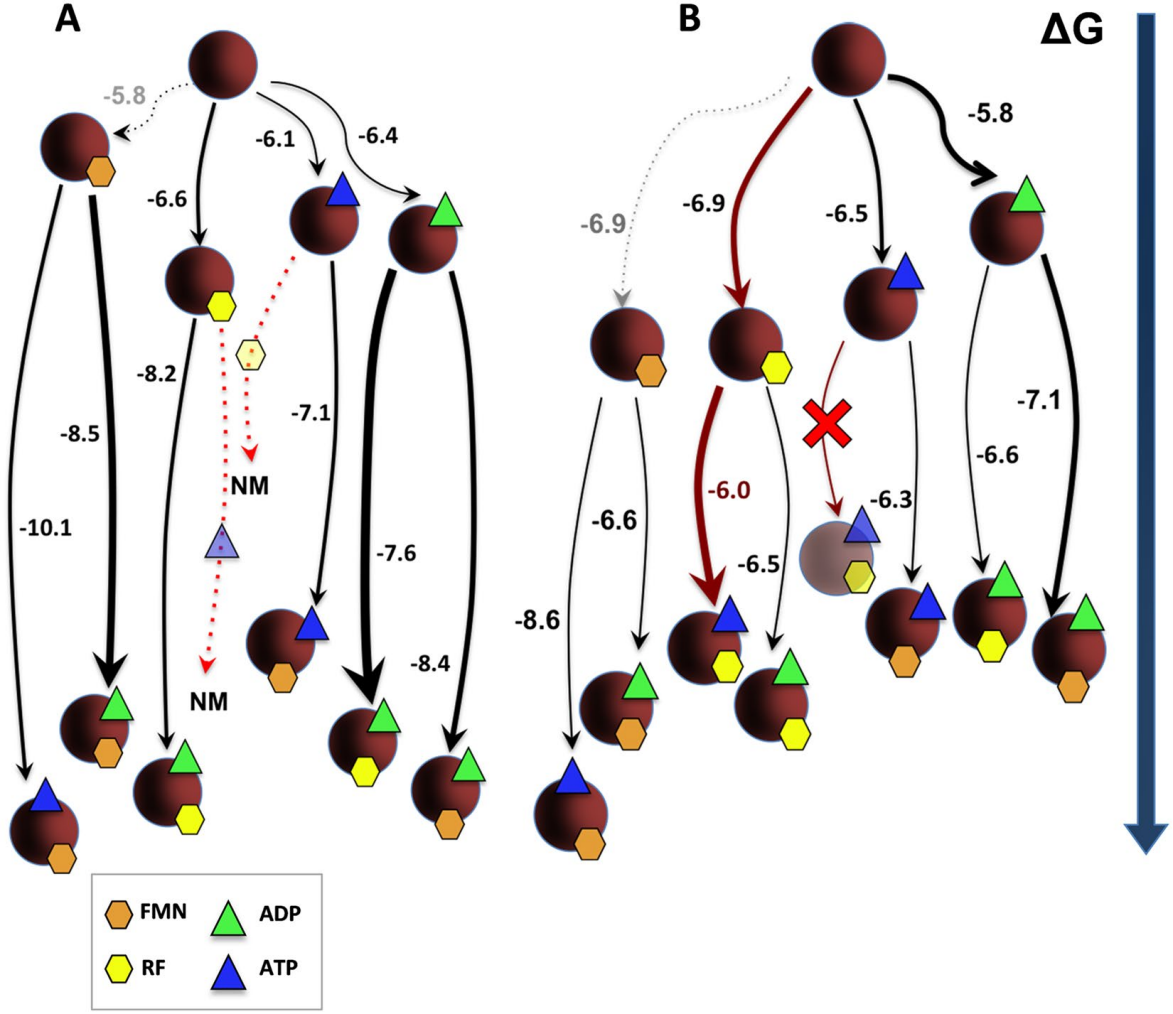
Gibbs free energy flow in the interaction between the RFK module and its ligands. Diagrams of the thermodynamics of interaction between the RFK module and different combinations of its ligands as obtained through ITC at 25 °C (**A**) in 20 mM PIPES and 0.8 mM MgCl_2_, pH 7.0 and (**B**) in 20 mM PIPES, pH 7.0. The RFK module is represented as maroon spheres, RF and FMN as yellow and orange hexagons, respectively, and ATP and ADP as blue and green triangles, respectively. Symbols for protein and ligands are shown with some level of transparency for those processes where calorimetric methods did not provide evidences of interaction. Numbers indicate the Δ*G* value for each titration. The thickness of arrows is proportional to the fraction of protein that is able to bind the ligand in each case, and the length of the lines is proportional to the Δ*G* value that is associated with each process. Dashed lines indicate processes that are not observed directly by ITC, but whose values were indirectly estimated using Eqn. 7 once the cooperativity coefficients were known. Red lines indicate the path leading to the simultaneous binding of both substrates required for RFK activity in the absence of the cation. NM accounts for processes with RF and ATP:Mg^2+^, where the heat exchanged in the catalytic reaction masks the interaction heat.

Figure 4 summarizes the free energy values (Δ*G*) that were determined for all possible combinations of the RFK module in binary and ternary complexes with different ligands, as well as the relative fraction of protein that was prone to interact, both in the presence (Fig. 4A) and in the absence of the divalent cation (Fig. 4B). All paths shown in the network compete with the one that leads to the catalytically competent complex by decreasing the amount of the RFK module that is available to bind the substrates of the reaction, RF and ATP:Mg^2+^. Paths with and without Mg^2+^ considerably differ in both the complex stability and its production probability (Fig. 4). Several remarkable facts need to be highlighted. We have no data for the reactive path containing RF and ATP:Mg^2+^ (the heat exchanged in the chemical reaction masks the binding enthalpy). We can include the path leading to a “pseudo-reactive” ternary complex that is formed in the absence of the cation and is produced by titration of the RFK module:RF binary complex with ATP (shown in maroon in Fig. 4B); however, in the absence of Mg^2+^, RF does not bind to the preformed binary RFK module:ATP complex. These data suggest that in the absence of the cation, the ATP substrate induces enzyme conformations that are not able to bind the RF substrate. All ternary interactions, as well as the single RF binding to the module, appear to be more favorable in the presence of Mg^2+^; furthermore, the cation also increases the protein fraction in the conformation that is competent to bind the ligands (as indicated by the thicker lines in Fig. 4A). Paths leading to transformation of binary complexes into ternary ones, despite not being competent for catalysis, are generally thermodynamically more favorable (as the more negative Δ*G* values show) than the “pseudo-reactive” one. It is also noticeable that, despite the fact that the direct binding of FMN to the RFK module is not detected by ITC (or by stopped-flow), the free energies of ANP binding to the RFK module are considerably more favorable when FMN is in the mixture. These data indicate that FMN might act as a slow binding ligand^31^ in its interaction with the RFK module in the absence of ANP and, in addition, suggest that there is binding cooperativity between the ligands.

### Adenine and flavin nucleotide ligands cooperate in their binding to the RFK module

**Figure 5:**
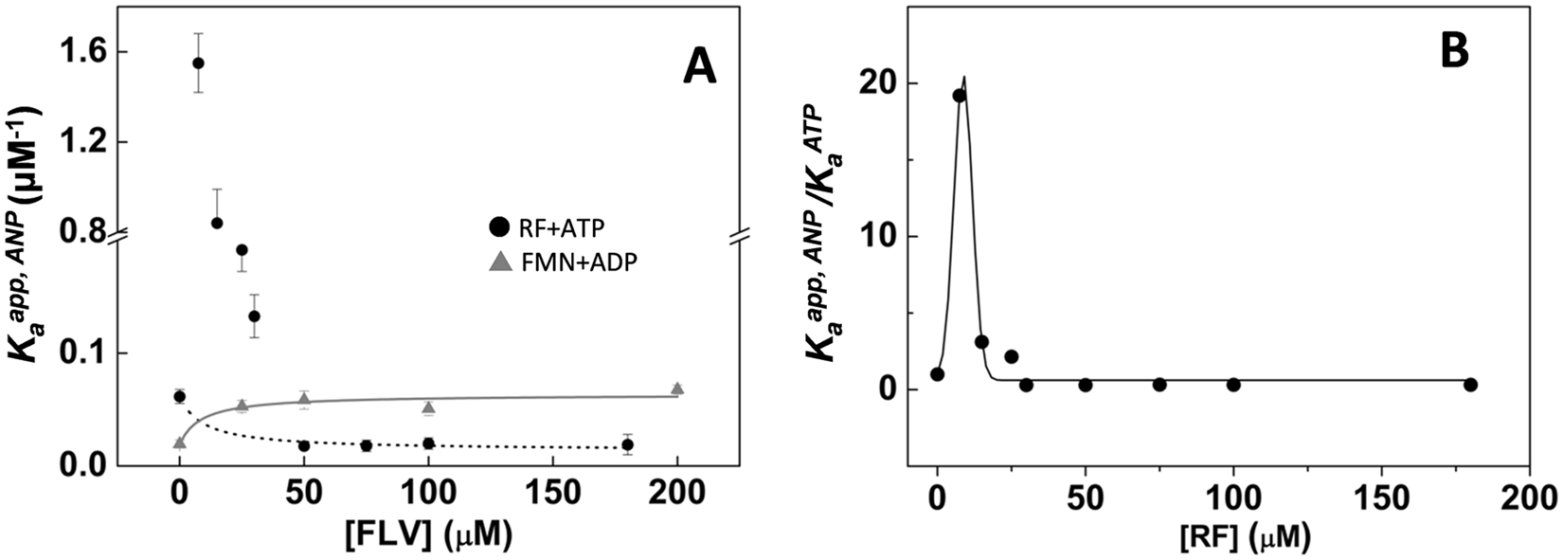
Binding cooperativity of the flavin and adenine nucleotide ligands to the RFK module. (**A**) Dependence of apparent ANP association constants, ${K}_{a}^{app,ANP}$, on the concentration of the flavin ligand for the titration with ATP of mixtures of the RFK module with different amounts of RF (●), as well as for the titration with ADP of mixtures of the RFK module with different amounts of FMN (▲). Lines show the fits to Eqn. 7 to determine the positive cooperativity between FMN and ADP (grey line) and the negative cooperativity between RF and ATP at RF concentration above 50 µM (dotted line)^32^. Points at lower RF concentrations are included to provide a view of ${K}_{a}^{app,ATP}$ evolution with RF concentration, but since fitting is not possible they are not connected to avoid misinterpretation. (**B**) Evolution of the ${K}_{a}^{app,ATP}/{K}_{a}^{ATP}$ ratio as a function of the RF concentration. All experiments were performed in 20 mM PIPES, pH 7.0 at 25 °C. Lines are included only as visual cues for the data evolution.

To prove the binding cooperativity between ligands, we performed sets of ITC experiments in which samples of the RFK module that contained different FLV ligand proportions were titrated with ANP ligands (Fig. SP4). These experiments allowed the determination of the apparent cooperativity constants for the ANP binding in the presence of the FLV ligand, ${K}_{a}^{app,ANP}$. Plots of ${K}_{a}^{app,ANP}$ *vs* FLV concentrations show different behaviors depending on the flavin and adenine nucleotide combination (Fig. 5A). FMN exhibits positive cooperativity with both ADP and ATP, as RF does with ADP. Although the RF substrate increases the protein’s affinity for ATP at RF concentrations up to 30 μM (maximum cooperativity at ≈7.5 μM), higher RF concentrations hinder ATP binding (Fig. 5). This decrease suggests that an excess of RF might block its binding.

We performed additional ANP titrations at saturating FLV concentrations, namely, 150 μM for RF or FMN and 7.5 μM RF for the ATP titration. The binding isotherms can be fit to a model that considers the reciprocal effect between the FLV and ANP ligands^32, 33^. In this way, cooperativity coefficients (α), the fraction of the protein sample that is able to bind the titrating ligand (N), and the enthalpy change associated with each process (Δ*h*), can be determined. The data are summarized in Table 3. The presence of the cation dramatically increases the binding cooperativity between flavin and adenine nucleotides (compare upper and lower parts of Table 3). It is also remarkable that the α values are larger than one, which indicates positive cooperativity, with the only exception being the combination of the RF and ATP substrates at high RF concentrations that showed negative cooperativity (α < 1). Therefore, although low concentrations of the RF substrate favor binding of the ATP substrate, an excess of RF has a negative impact on ATP binding (Figs 5B and SP4 and Table 3). The products of the reaction (FMN and ADP) always favor binding of the other substrates and products (Fig. 5A and Table 3). Moreover, the presence of either Mg^2+^ or ATP considerably enhances cooperativity, with the cooperativity for the FMN-ATP:Mg^2+^ combination being particularly large (Table 3). This later observation probably relates to the fact that in the absence of ATP, FMN seems to act as a slow binding ligand, which makes its binding to the RFK module not detectable by fast kinetics or ITC methods. Once the α values are known, the experimental ${K}_{a}^{app,ANP}$ and Eqn. 7 can also be used to predict the association constant for FMN binding to the free RFK module, ${K}_{a}^{FMN}$, as well as the corresponding Δ*G* values presented in Fig. 4 (gray dotted lines).

**Table 3:** Cooperativity coefficients (α) for the binding of different combinations of FLV and ANP ligands to the RFK module.

	ligands	α	N	Δ*h* (kcal/mol)
0.8 mM MgCl_2_	FMN-ADP	56 ± 4	0.6 ± 0.01	−8.1 ± 1.9
	FMN-ATP	920 ± 70	0.2 ± 0.01	−22 ± 1
	RF-ADP	24 ± 2	0.3 ± 0.01	−7.1 ± 0.3
0 mM MgCl_2_	FMN-ADP	3.8 ± 0.2	0.2 ± 0.01	−9.7 ± 0.8
	FMN-ATP	15 ± 1	0.02 ± 0.003	−30 ± 1
	RF-ADP	3.3 ± 0.1	0.2 ± 0.02	−20 ± 1
	RF-ATP^a^	19 ± 6	0.5 ± 0.06	−8.1 ± 1.1
	RF-ATP^b^	0.8 ± 0.1	0.8 ± 0.07	−15 ± 4

Experiments were carried out at 25 °C in PIPES 20 mM and 0.8 mM MgCl_2_, pH 7.0, as well as in the same buffer without MgCl_2_. The variable α represents the cooperativity coefficient between each pair of ligands, N the fraction of total protein able to bind the titrating ligand, and Δ*h* the enthalpy change associated with each process. Errors were generally assumed to be larger than the standard deviation between replicates and the numerical error after fitting analysis.

^a^Data from the titration at the maximum of cooperativity, 7.5 μM RF.

^b^Data from the titration at 180 μM RF.

## Discussion

### The collective binding of adenine and flavin nucleotides induces important conformational changes in the *Ca*FADS RFK module

**Figure 6:**
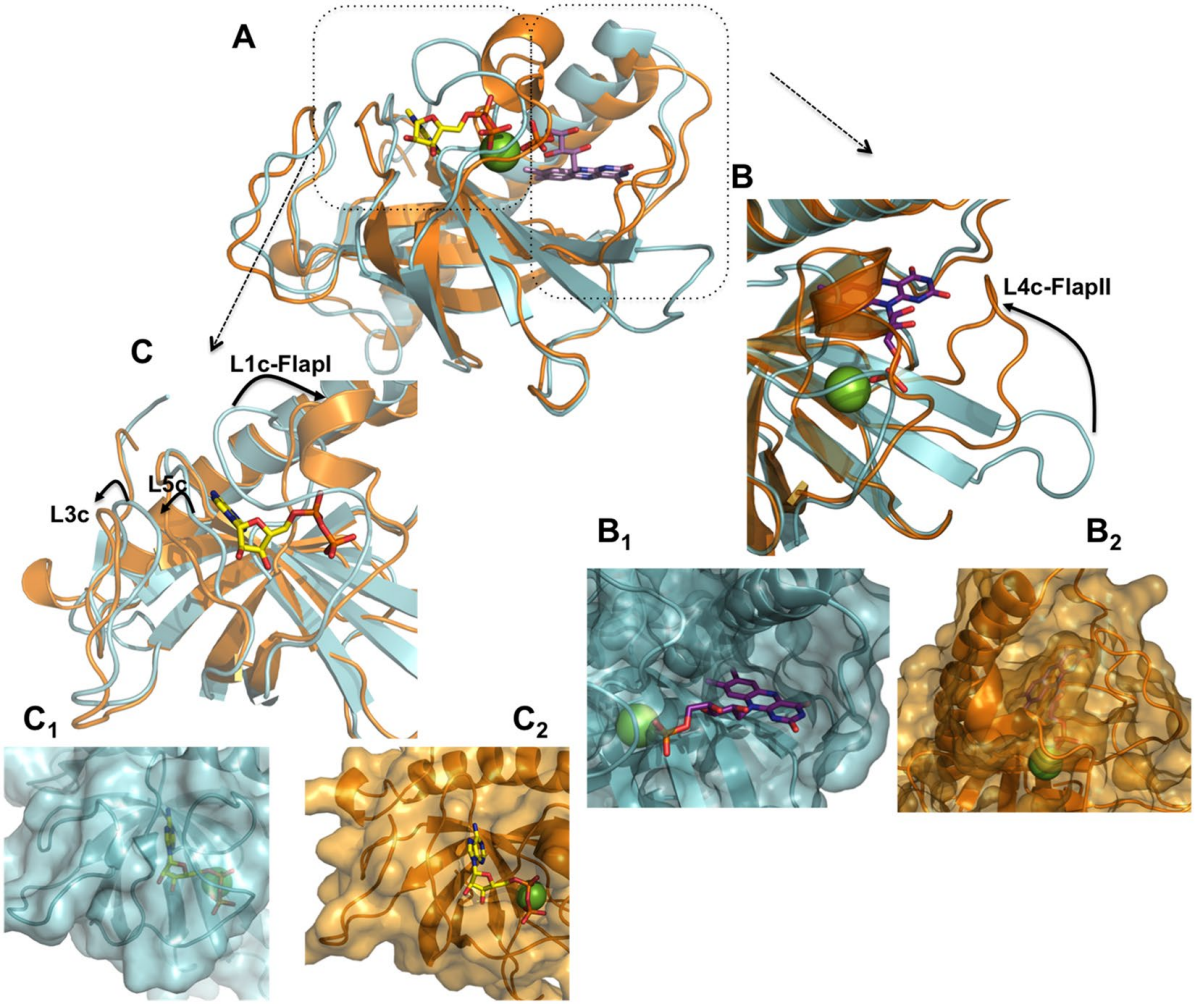
Structure of the RFK module of *Ca*FADS. Crystallographic structures of the RFK module when free (blue, PDB 2x 0 k) and in the ternary complex with the FMN and ADP:Mg^2+^ products (orange, PDB 5a89). (**A**) Superposition of both structures. (**B**) Detail of the flavin nucleotide-binding cavity. B_1_ shows the open conformation of the cavity in the free enzyme, where the phosphate of FMN and the Mg^2+^ cation will find their binding site blocked by residues of L1c. B_2_ shows the enclosure of the flavin ring by L4c in the ternary complex. (**C**) Detail of the adenine nucleotide-binding cavity. C_1_ shows the closed conformation of the ANP-binding site in the free enzyme, where the phosphates of ANP and the Mg^2+^ cation will find their binding site blocked by residues of L1c. C_2_ shows the open conformation of this cavity with ADP:Mg^2+^ bound in the ternary complex. In all panels, the ligands correspond to those in the 5a89 structure. ADP and FMN ligands are shown as sticks and are CPK colored with carbons in yellow and purple respectively, and the Mg^2+^ cation is shown as a green sphere.

Analysis of our data in the context of the available structures for the RFK module, free and in the FMN:ADP:Mg^2+^ non-productive ternary complex (Fig. 6)^18^, allows to infer new insights about the binding and dissociation of substrates and products during the course of the enzyme catalytic cycle. Although changes in the RF fluorescence are not detected upon its fast mixing with the RFK module, ITC experiments reveal that RF is able to interact with the RFK module with moderate affinity (Table SP1). Such RF binding is consistent with the wide-open cavity that the free RFK module shows at the ribityl and isoalloxazine binding sites (Fig. 6, panel B_1_). RF might be recognized by this cavity at the RFK module, although our biophysical data indicate that its isoalloxazine ring is not enclosed within the protein by the displacement of Flap-II. In contrast, in the free protein crystal structure, several loops and sidechains block the adenine nucleotide-binding site, including the pocket for the phosphate groups (Fig. 6, panels B_1_ and C_1_)^14^. ATP (as well as ADP) reduces the access of the RF isoalloxazine to the solvent (Fig. 3 and Table SP1), suggesting its competent binding. This result agrees with the formation of a ternary complex identical to that in the RFK module:FMN:ADP:Mg^2+^ crystal structure (Fig. 6)^18^. Comparing these two structures shows that significant rearrangements have occurred. They include displacements of loops L3c, L5c and L1c-FlapI to allow the binding of the adenine nucleotide, as well as a noticeable change in the conformation of loop L4c-FlapII, which encloses the isoalloxazine ring inside the protein (Fig. 6). The large unfavorable entropic and favorable enthalpic contributions for ATP binding to the RFK module:RF mixture, as well as for RF binding to the RFK module:ADP mixture (Table SP1 and Fig. SP3), further support the idea that such rearrangements occur upon concerted ligand binding. Therefore, it appears that large conformational changes occur in the transformation of binary interactions into ternary complexes^18^.

### Mg^2+^and the concerted fit of substrates to achieve the catalytically competent complex

**Figure 7:**
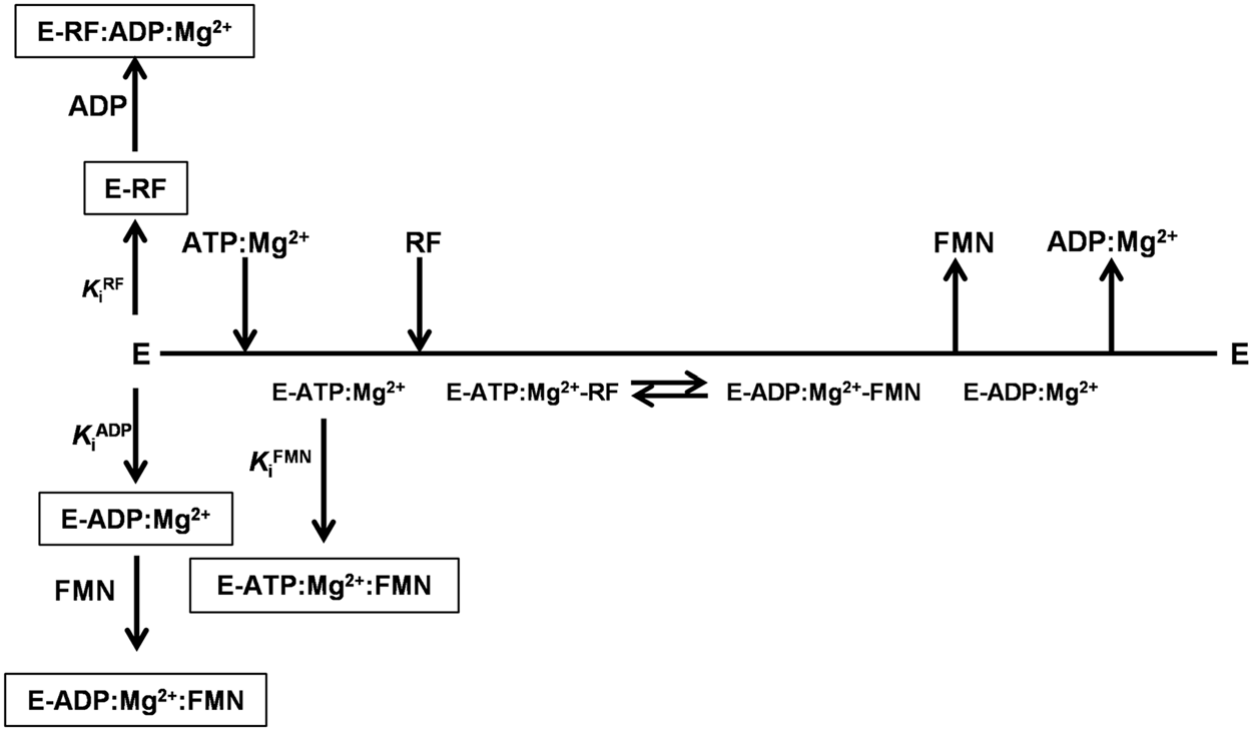
Cleland representation of the kinetic mechanism for the transformation of RF into FMN by the RFK module of *Ca*FADS. The different inhibition processes that were identified are summarized.

Fast kinetic methods allowed the determination of a dissociation constant in the low micromolar range for the productive RFK module:RF:ATP:Mg^2+^ ternary interaction (Table 2). This interaction, which is not thermodynamically detected in the absence of the cation, suggests that previous ATP:Mg^2+^ binding is required for the binding of competent RF to the RFK module (Fig. 7). Three observations support this mechanism: (i) the cation-mediated conformational changes that enclose the flavin ring and fix the ribityl end in a geometry that is competent for its phosphorylation^18^, (ii) the previously reported molecular dynamics simulations, which show how phosphates that are Mg^2+^-bridged to the active site promote the opening of the adenine-binding site^18^, and (iii) the fact that RF is not able to interact with the preformed RFK module:ATP complex in the absence of the cation but is transformed into FMN when mixed with the RFK module:ATP:Mg^2+^ mixture. Collectively, these results indicate that a concerted binding of ATP:Mg^2+^ and RF is necessary to achieve the substrates induced fit in the catalytically competent conformation.

### The RFK activity is highly regulated by inhibition by substrates and products

The binding of the substrates of the RFK reaction showed positive cooperativity at low RF concentrations, while higher RF concentrations hindered ATP binding (Fig. 5 and Table 3). This fact points to the formation of non-productive RFK module:RF interactions as the main cause for RF substrate inhibition (Figs 2A and 7)^20^. Fast kinetic data indicate that the opening of the flavin-binding cavity to release the flavin products is the process that is prevented (Figs 3 and SP2), whereas cooperative binding data (Table 3) point to an increase in the protein occupation by ligands as the RF concentration increases. Such observations are consistent with an excess of RF inducing binding modes that prevent conformational reorganizations that are required for product release to initiate a new catalytic cycle.

The FMN and ADP products, accumulating as the RFK reaction proceeds, also inhibit the RFK reaction through different mechanisms (Fig. 7). FMN is not able to bind quickly enough to inhibit the free RFK module, but it behaves as an uncompetitive inhibitor that binds to the preformed ATP-enzyme complex and forms a highly stable dead-end complex (Table 1 and Table SP1 and Figs 4 and SP3). Apparently, the phosphate group at the ribityl end in the FMN product might prevent the placement and enclosure of this cofactor (Fig. 6). When combining FMN and ATP with the RFK module, fast kinetic binding rates show a biphasic dependence on the FMN concentration. The faster rates are produced in the lower range of concentrations, but rates rapidly decrease with increasing FMN concentration (Fig. 3E). The combination of these ligands also produces a huge cooperativity (Table 3) and a very stable non-productive ternary complex (dead-end complex), while FMN binding to the preformed enzyme:ATP binary complex is considerably less stable (Fig. 4). Considering that FMN and ATP are the substrates of the reaction that is catalyzed by the *Ca*FADS FMNAT module, it is likely that under situations requiring FAD synthesis, the dead-end ternary complexes might facilitate channeling of FMN and ATP to the FMNAT module. This possibility must be considered within the framework of the head-to-tail RFK-to-FMNAT arrangement between protomers within trimers in the dimer-of-trimers structure that has been reported for the complete enzyme^14^. ADP acts as competitive inhibitor, competing with ATP for the same binding site (Fig. 7). The higher *K*_*m*_/*K*_*i*_ ratio for FMN relative to ADP, ≈5 and ≈2.4, respectively, points to the flavin product as a more potent inhibitor. This fact, together with the negative cooperativity between RF and ATP at high RF concentrations (Table 3), can explain why inhibition of the *Ca*FADS RFK activity by ADP has not been previously detected.

Our data suggest that FMN and ADP are able to inhibit the RFK reaction through the formation of different non-productive ternary complexes (Fig. 4). The time-dependent increase of flavin fluorescence that was observed in samples containing ATP (not observed for ADP) after ternary complex formation (Fig. 3) suggests dissimilar organizations of complexes with the ANP substrate and product. The presence of these different complexes is also supported by differences in their thermodynamic binding parameters (Table SP1 and Fig. 4). Noticeably, the binding of substrates is slower than the formation of all the non-productive combinations of adenine and flavin nucleotides (Fig. 3E and Table 2), in addition to being less thermodynamically favorable. Therefore, all combinations of ternary complexes might compete with the catalytic complex as the reaction progresses, with the ternary non-productive complexes that contain the ADP:Mg^2+^ product being particularly populated (thicker lines in Figs 4A and 7). The population of these non-productive complexes reduces the number of enzyme molecules that are available to initiate a new catalytic RFK cycle. Moreover as higher is the concentration of one of the ligands in the ternary non-productive complexes, the stronger that the second ligand binds (Fig. 5 and Table 3). Thus, binding of substrates during the RFK reaction is less favorable when the products accumulate.

### FMN synthesis is a key process that requires tight regulation

FMN production by *Ca*FADS is highly regulated by substrate and product inhibition of its RFK activity to avoid overproduction even though the RF substrate might transiently increases in the media, where usually the amount of RF is considerably lower than that of FMN and particularly of FAD (either free or as part of flavoproteins)^34, 35^. Moreover, ADP and the ATP nucleotides bind similarly to the RFK module, while the binding pockets for RF and FMN are dissimilar. In addition, we must keep in mind that the FMNAT activity at the N-terminus module of the *Ca*FADS does not show any type of regulation by substrate or product inhibition^19^. Considering that selective inhibition of key enzymes is a common tool to regulate metabolic pathways^25–27, 36^, inhibition of RFK activity by its substrates and products appears to be a useful tool to regulate the availability of both FMN and FAD in *C*. *ammoniagenes*. Most known bacteria are able to synthesize their own RF^37, 38^, but at the same time are also able to take it up from the environment employing transporters^4, 38–40^. Therefore, mechanisms avoiding FMN and FAD overproduction when RF concentration transiently increases must be relevant. Although the study of additional family members is required to evaluate the generality of this mechanism, the data available for the few prokaryotic FADSs so far characterized suggest that strategies other from RFK substrate inhibition are used to regulate FMN and FAD production. Such differences among family members might also provide a framework to design selective compounds that target prokaryotic FADS in the treatment of diverse infectious diseases. Past ten years have seen significant advances in our knowledge of enzymes of the RFK and FMNAT families from different kingdoms, opening questions regarding their biochemical and mechanistic behaviour as well as their function in the regulation of flavin metabolism. Answering to them will provide interesting challenges for future research as well as for beneficial applications.

## Conclusion

Prokaryotic FADSs have a major functional role in providing flavin cofactors to the cellular flavoproteome while also promoting flavin and flavoprotein homeostasis. This study sheds light on the mechanism of the RFK activity of *Ca*FADS and on its regulation by substrates and products through a better understanding of their inhibitory effects and the kinetics and the thermodynamics of their binding. Our results lead us to conclude that the collective binding of adenine and flavin nucleotides induces important conformational changes in the *Ca*FADS RFK module. In this mechanism, Mg^2+^ and the concerted fit of substrates are required to achieve a catalytically competent geometry. In addition, all possible combinations of adenine and flavin nucleotide substrates and products produce the inhibition of RFK activity at the RFK module of *Ca*FADS. In fact, production of all possible non-competent combinations competes with the catalytically competent configuration, both kinetically and thermodynamically. This careful regulation is surely not odd considering the plethora of processes in which FMN and FAD act as flavoprotein cofactors, highlighting the importance of producing FMN in appropriate amounts. In agreement with the selective inhibition of key enzymes being a common tool to regulate metabolic pathways, inhibition of the RFK activity provides an intricate regulatory mechanism that allows the suitable production of flavins according to the *C*. *ammoniagenes* cellular needs. Thus, further investigations in other RFKs and FMNATs is required to evaluate the relevance of *Ca*FADS as general model and to identify in which extension differences among family members can relate to dissimilarities in the regulation of FMN and FAD biosynthesis in different species.

## Materials and Methods

### Cloning, expression and purification

The C-terminal domain was individually cloned (pET28a-Δ(1–182)FADS) using the protocol described for WT *Ca*FADS with the forward primer 5′-CCAACTGGGCCATGGGGCGGCAC-3′ and the reverse primer previously used for the full length protein cloning. The forward primer used to clone the C-terminal domain incorporates the start codon ATG in a *NcoI* site substituting for Leu183^12^. The pET28a-Δ(1–182)FADS plasmid was introduced into the *E*. *coli* BL21(DE3) strain, to overexpress the RFK module of *Ca*FADS and then purify the protein by following the protocols that have been previously described^16^. The pure protein was concentrated in 20 mM PIPES, pH 7.0, and quantified using its theoretical molar extinction coefficient at 280 nm, 14.44 mM^−1^·cm^−1^. All experiments were carried out under oxygen atmosphere, since in *Ca*FADS activities have been shown independent of the flavins redox state^19, 20^.

### Steady-state RFK activity

The RFK activity of the RFK module was measured at 25 °C in 500 µL of 20 mM PIPES and 0.8 mM MgCl_2_, pH 7.0, containing variable concentrations of RF (0.5–45 µM) and ATP (10–500 µM ATP), as previously reported^19, 20^. The inhibitory effect of the reaction products was evaluated in reaction mixtures containing 1–30 μM RF and 350 μM ATP at different FMN concentrations (0–20 μM) when analyzing the FMN inhibitory effect, or 10–350 μM ATP and 5 μM RF at variable ADP concentrations (0–400 μM) when examining inhibition by ADP. In all cases, reactions were initiated by addition of the enzyme to a final concentration of ≈40 nM, which was followed by 1 min of incubation, and the reactions were stopped by boiling the samples at 100 °C for 5 min as previously described^19, 20^. The flavin composition of the supernatant was analyzed using an Alliance HPLC system (*Waters*) equipped with a 2707 autosampler and an HSST3 column (4.6 × 50 mm, 3.5 mm; *Waters*) preceded by a precolumn (4.6 × 20 mm, 3.5 mm; *Waters*) as previously described^19, 20^. Flavin concentrations (RF or FMN) were quantified using their corresponding standard curves, and the observed steady-state rates (*v*_0_) were determined in units of nmoles of flavin transformed *per* min *per* nmol of enzyme (*v*_0_/[*e*])^19, 20^. All the experiments were carried out in triplicate.

Kinetic data were obtained at different ATP concentrations and 5 µM RF (concentration for which the free enzyme shows ∼80% of its maximal apparent activity before the maximum experimentally detected, since, as previously reported, the high inhibition by RF prevents to work with saturating concentrations of RF^19, 20^) and were fit to the Michaelis-Menten model to obtain the Michaelis-Menten (*K*_*m*_) and catalytic rate (*k*_*cat*_) constants. Kinetic data obtained when varying RF concentrations at saturating ATP concentrations showed inhibition by an excess of the RF substrate; therefore, a model describing the substrate inhibition effect produced in bi-substrate enzyme kinetics was used to interpret the experimental data according to the following equation:

1 $$\frac{{v}_{0}}{[e]}=\frac{{k}_{{\rm{cat}}}\,[{\rm{S}}]}{{K}_{{\rm{m}}}^{{\rm{s}}}+[{\rm{S}}](1+\frac{[{\rm{S}}]}{{K}_{i}^{s}})}$$

where *v*_0_ is the determined observed rate constant at each experimental condition, [*e*] is the enzyme concentration in the assay, [S] is the concentration of the inhibitory substrate, and *K*_i_^S^ is the substrate inhibition constant. In general, the strong inhibition by the RF substrate prevented the determination of limiting catalytic and Michaelis constants, and data obtained in this study correspond to apparent constants ^app^ *k*_cat_ and ^app^ *K*_m_^20, 21, 41^. The inhibition mechanisms of the FMN and ADP products were identified by evaluating the effect of both products on the *K*_*m*_ and *k*_*cat*_ values obtained by the independent fitting of data sets to the Michaelis-Menten model. Additionally, Dixon plots (1/(*v*_0_/[*e*]) *vs* [I]) and Cornish-Bowden representations ([S]/(*v*_0_/[*e*]) *vs* [I]) were used to reveal the type of inhibition^42, 43^. Finally, the different data sets were globally fit using the Lineweaver-Burk equations for competitive and uncompetitive inhibition^42^,

2 $$\frac{[e]}{{v}_{0}}=\frac{[1+\frac{[I]}{{K}_{i}^{P}}]{K}_{m}}{{k}_{{\rm{cat}}}[S]}+\frac{1}{{k}_{{\rm{cat}}}}$$

3 $$\frac{[e]}{{v}_{0}}=\frac{1+\frac{[I]}{{K}_{i}^{P}}}{{k}_{{\rm{cat}}}}+\frac{{K}_{m}}{{k}_{{\rm{cat}}}[S]}$$

where [S] and [I] represent the concentrations of substrate and product inhibitor, respectively, and ${K}_{i}^{P}$ is the corresponding product inhibition constant^42^. Estimated errors in *k*_cat_, *K*_m_ and *K*_*i*_ values are in general within ± 15% of their values, but when RF produces strong dead-end inhibition, errors can considerably increase due to the similarity between ${K}_{i}^{RF}$ and ${K}_{m}^{RF}$ values.

### Pre-steady-state kinetics

Pre-steady-state kinetic experiments were performed using stopped-flow spectroscopy on an *Applied Photophysics* SX17.MV spectrophotometer using the Xscan software (*Applied Photophysics Ltd*.). The fluorescence of the flavins was measured in a continuous assay with an excitation wavelength of 445 nm, and the emission was recovered using a >530 nm cut-off filter. The selected voltage was 350 V, and 800 points were taken in every measurement. Measurements were carried out at 25 °C in 20 mM PIPES and 0.8 mM MgCl_2_, pH 7.0. The RFK module (0.2 μM) was mixed with reaction samples and the concentration of the flavin (FLV, herein referring to either RF or FMN) was varied from 0.5–5 μM both in the absence and under saturating concentrations of adenine nucleotides (ANP, ATP or ADP ligands). Control experiments, recorded in the same buffer but in the absence of MgCl_2_, generally produced similar profiles but considerably smaller amplitudes in the fluorescence changes. All the concentrations that are indicated are the final ones in the reaction cell. Every kinetic trace was recorded until getting at least three reproducible traces.

Kinetic traces of changes in fluorescence that showed exponential behavior were fit to exponential equations (Eqn. 4) in which each exponential term describes a different spectroscopic process. When a particular process was not finished within the measurement timeframe, a linear correction term (b) was added to the equation to account for the unfinished process (Eqn. 5).

4 $$y=\sum {A}_{i}{e}^{-{k}_{obs,i}t}$$

5 $$y=\sum {A}_{i}{e}^{-{k}_{obs,i}t}+bt$$

In these equations, *A*_*i*_ and *k*_*obs*,*i*_ are the amplitude and the observed rate constant, respectively, for each of the processes (*i*) that contribute to the overall time-dependent fluorescence change for the particular conditions of each experiment.

When *k*_*obs*_ values showed a linear dependence on the flavin concentration, they were fit to a one-step model that accounts for the equilibrium of enzyme-flavin complex, whose kinetics can be represented by the following equation

6 $${k}_{obs}={k}_{on}[FLV]+{k}_{off}$$

where *k*_*on*_ and *k*_*off*_ are the kinetic constants for complex formation and dissociation, respectively.

Due to the deterioration that flavins undergo when they are exposed to light, we also evaluated the FLV photobleaching decay during measurements. Under our experimental conditions, RF and FMN fluorescence decay linearly with time (Fig. SP1A). Slopes for these kinetic traces linearly decrease with the flavin concentration, with slopes corresponding to photobleaching rates of 4.6 · 10^−3^ ± 1.8 · 10^−4^ min^−1^ and 4.1 · 10^−3^ ± 1.8 · 10^−4^ min^−1^ for RF and FMN, respectively (Fig. SP1B).

### Isothermal titration calorimetry

Isothermal titration calorimetry (ITC) experiments were performed to elucidate the order in which ligands bind to the enzyme, as well as the binding inhibition effects from the thermodynamic point of view. Experiments were carried out in an AutoITC200 (*MicroCal*) that was thermostated at 25 °C. Typically, 180 μM RF, 350 μM ATP or ADP, and 250 μM FMN solutions were used to titrate the RFK module (≈25 μM) in a 200-μL cell. Titrations with FLV ligands of mixtures that contained the enzyme prebound with the ANP ligand were also carried out, as well as ANP titrations of mixtures of the enzyme saturated with FLV ligands. In these experiments, the titration was performed by stepwise injecting the titrating ligand. Up to 19 injections of 2 μL each were added to the calorimetric sample cell and mixed at a stirring speed of 1000 r.p.m. Ligands and protein were dissolved in 20 mM PIPES, pH 7.0, either in the presence or absence of 0.8 mM MgCl_2_, and degassed prior to titration. The association constant (*K*_*a*_), the enthalpy change (Δ*H*) and the binding stoichiometry (N) were obtained through a nonlinear least squares regression of the data using a homemade model for one or two independent binding sites, which was implemented in Origin 7.0 (*OriginLab*) as previously described^16, 20^. The entropic contribution (-TΔ*S*), the Gibbs free energy (Δ*G*) and the dissociation constant (*K*_*d*_) were obtained through essential thermodynamic equations.

To determine the cooperativity coefficients (α) describing the interactions between the ANP and the FLV ligands, a set of six additional experiments was carried out. Mixtures containing the RFK module at six different FLV concentrations were titrated with ANP ligands. These experiments allowed the determination of the apparent association constants for the adenine nucleotide ligand, ${K}_{a}^{app,ANP}$, at each particular concentration of the FLV ligand mixed with the protein in the calorimetric cell. The data were fit to the equation describing the dependency of ${K}_{a}^{app,ANP}$ as a function of the FLV concentration and α (cooperativity constant for the heterotropic interaction between ANP and FLV)

7 $${K}_{a}^{app,ANP}={K}_{a}^{ANP}\frac{1+\alpha \,{K}_{a}^{FLV}\,[FLV]}{1+{K}_{a}^{FLV}\,[FLV]}$$

where ${K}_{a}^{ANP}$ is the association constant for ANP, ${K}_{a}^{FLV}$ is the association constant for FLV, and [FLV] is the concentration of flavin in the calorimetric cell. Additionally, particular titrations performed at saturating FLV concentration (100 µM) were fit to a homemade model that considers the influence of the FLV on the protein affinity for the ANP^32, 33^, yielding similar α values.

Experiments were performed in triplicate. The errors considered in the measured parameters (±15% in *K*_d_ and *K*_a_ values, ±0.3 kcal·mol^−1^ in Δ*G*, Δ*H* and −TΔ*S* and ± 20% in α) were assumed to be larger than the standard deviation between replicates and the numerical error after fitting analysis.

## Electronic supplementary material

Supplementary Information
