# Supplementary material for: Kinetics and thermodynamics of the protein-ligand interactions in the riboflavin kinase activity of the FAD synthetase from Corynebacterium ammoniagenes
Source: Sci Rep. 2017 Aug 4;7:7281. doi: 10.1038/s41598-017-07875-5 (PMC5544777; doi:10.1038/s41598-017-07875-5)
Supplement: Supplementary file 1 — Supplementary Information [file 41598_2017_7875_MOESM1_ESM.pdf]

Kinetics and thermodynamics of the protein-ligand interactions in the riboflavin kinase activity of the FAD synthetase from *Corynebacterium ammoniagenes*

María Sebastián[a], Ana Serrano[a,1], Adrián Velázquez-Campoy[a,b,c], and Milagros Medina[a,‡]

[a] Department of Biochemistry and Molecular and Cellular Biology, Faculty of Sciences, and Institute of Biocomputation and Physics of Complex Systems (Joint Units: BIFI-IQFR and GBsC-CSIC), University of Zaragoza, Spain

[b] ARAID Foundation, Diputación General de Aragón, Spain

[c]Aragon Institute for Health Research (IIS Aragon), Zaragoza, 50009, Spain

[‡]Correspondence to: Milagros Medina, Departamento de Bioquímica y Biología Molecular y Celular. Facultad de Ciencias, Universidad de Zaragoza. Pedro Cerbuna, 12. 50009 Zaragoza, Spain. Tel: +34976762476; Fax: +34976762123. E-mail: mmedina@unizar.es

[1]Present address: Centro de Investigaciones Biológicas, CSIC, Ramiro de Maeztu 9, E-28040 Madrid, Spain

RUNNING TITLE: **The riboflavin kinase mechanism of FAD synthetase from *Corynebacterium ammoniagenes***

SUPLEMENTAL FIGURES

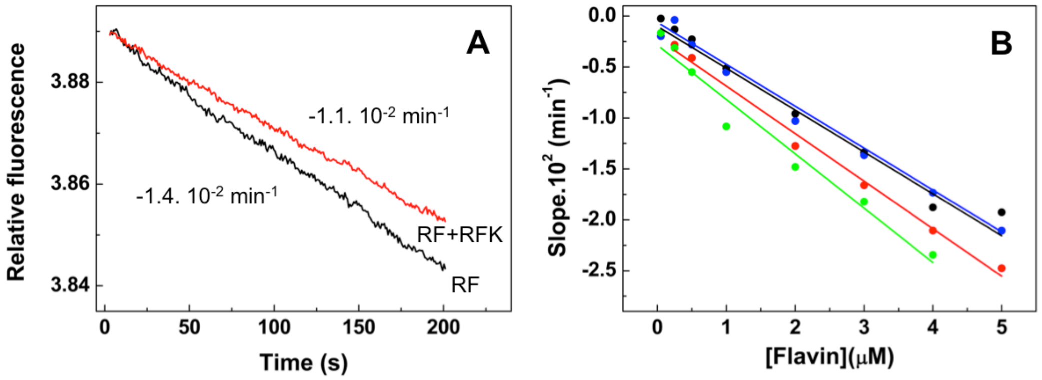

Figure SP1. Presteady-state kinetic analysis of flavins photodecay and of their binding to the RFK module of *Ca*FADS. (A) Kinetic traces of the evolution of the RF fluorescence (1 µM) in the stopped-flow equipment follow zero order decays both in the absence (black line) and presence (red line) of the RFK module (0.2 µM). (B) Slopes of linear fluorescence decays on the flavin concentration for samples containing RF (black), FMN (blue), as well as RF (red) and FMN (green) in the presence of the RFK module. All mixtures show linear dependences on flavin concentrations with similar negative slopes. They, therefore, indicate that increasing flavin concentrations protect from photobleaching during measurements. Experiments were performed in 20 mM PIPES, 0.8 mM $MgCl_2$, pH 7.0 at 25 °C.

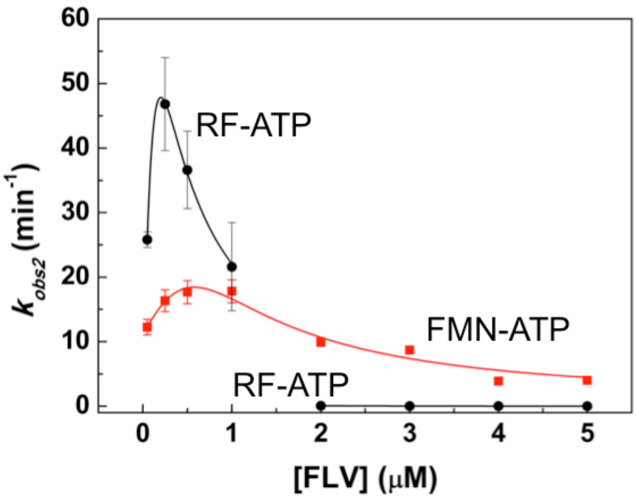

Figure SP2. (A) Evolution of k_{obs2} as a function of the FMN concentration for reaction mixtures of the RFK module with FMN+ATP (red) and with RF+ATP (black) follow a biphasic behavior with maximum ~0.5 µM FMN and ~0.25 µM RF. All the experiments were carried out in the stopped-flow equipment at 25°C in 20 mM PIPES and, 0.8 mM $MgCl_2$, pH 7.0 with 0.2 µM of the RFK module. All concentrations that are indicated are final concentrations in the mixing cell. Other conditions as in Figure 3.

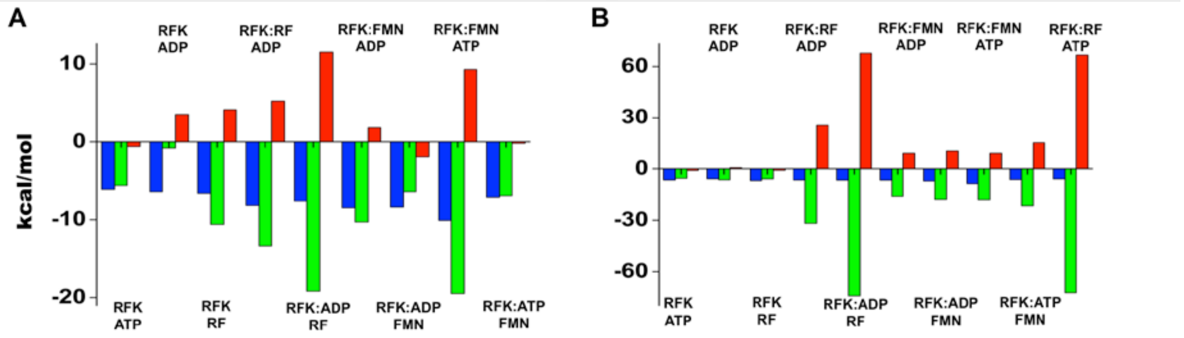

Figure SP3. Thermodynamic dissection of the interaction of different ligands with either the free RFK module, or with the preformed mixtures of the RFK module with different ligands (RFK:X). Parameters obtained (A) in the presence of 0.8 mM $MgCl_2$ and (B) in the absence of $MgCl_2$. Experiments were carried out in 20 mM PIPES, pH 7.0, at 25°C. The binding Gibbs energy (Δ*G*), enthalpy (Δ*H*), and entropy (-TΔ*S*) contributions to the binding are represented in blue, green and red bars, respectively.

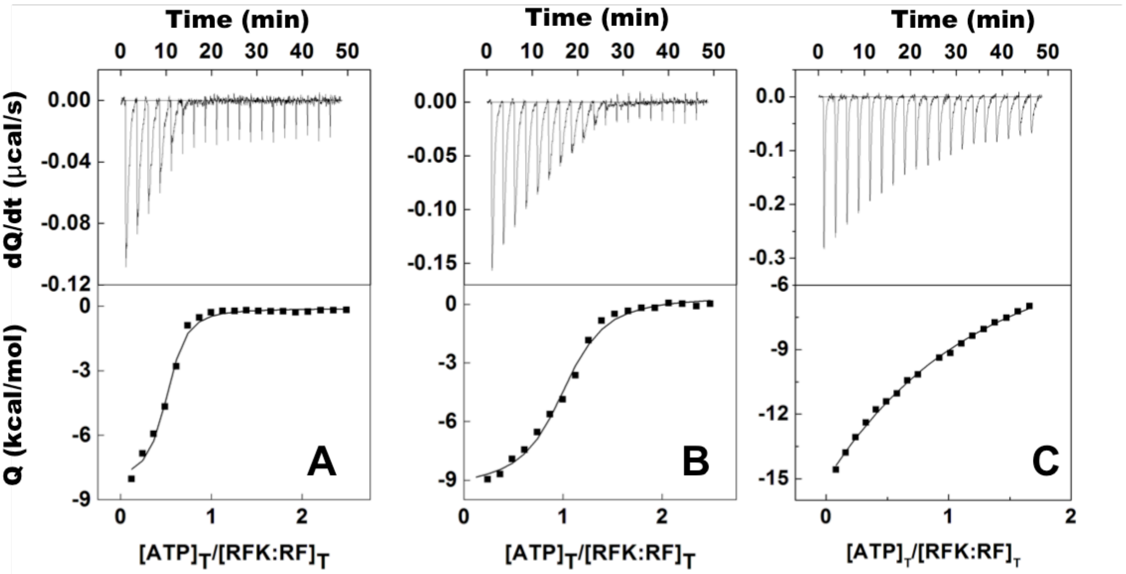

Figure SP4. Calorimetric titrations with ATP of mixtures containing different ratios of the RFK module and the RF ligand (RFK:X). Experiments were performed at (A) 7.5, (B) 15, and (C) 180 µM of RF with 25 µM RFK module in 20 mM PIPES, pH 7.0 at 25 ºC. Data were fit to a home-derived model for a single binding site. Within each panel, upper charts show thermograms for the different interactions and the lower panels the corresponding binding isotherms with integrated heats.

SUPLEMENTAL TABLE

Table SP1. Thermodynamic interaction parameters for the combination of the RFK module with different ligands as obtained through ITC. Column named initial mixture indicates the starting composition of the calorimetric cell. Titrations were carried out at 25ºC in 20 mM PIPES, 0.8 mM $MgCl_2$, pH 7.0 (upper table) and in 20 mM PIPES, pH 7.0 (lower table). Errors in Δ*G*, Δ*H* and –TΔ*S* were estimated in ±0.3 kcal/mol, taken in general larger than the standard deviation between three replicates and the numerical error after fitting analysis.

0.8 mM $MgCl_2$					
initial mixture	ligand	K_d (µM)	Δ*G* (kcal/mol)	Δ*H* (kcal/mol)	-*T* Δ*S* (kcal/mol)
RFK	ATP	28.7	-6.1	-5.6	-0.6
RFK	ADP	22.1	-6.4	-9.8	3.5
RFK	RF	15.2	-6.6	-10.6	4.1
RFK	FMN	n.d.[a]	n.d.[a]	n.d.[a]	n.d.[a]
RFK:RF	ADP	1.0	-8.2	-13.4	5.2
RFK:RF	ATP	n.m.[b]	n.m.[b]	n.m.[b]	n.m.[b]
RFK:FMN	ADP	0.6	-8.5	-10.3	1.8
RFK:FMN	ATP	0.04	-10.1	-19.5	9.3
RFK:ATP	FMN	6.6	-7.1	-6.9	-0.2
RFK:ATP	RF	n.m.[b]	n.m.[b]	n.m.[b]	n.m.[b]
RFK:ADP	FMN	0.8	-8.4	-6.4	-1.9
RFK:ADP	RF	2.3	-7.6	-19.2	11.5

0.0 mM $MgCl_2$					
initial mixture	ligand	K_d (µM)	Δ*G* (kcal/mol)	Δ*H* (kcal/mol)	-*T* Δ*S* (kcal/mol)
RFK	ATP	16.3	-6.5	-5.5	-1.0
RFK	ADP	51.4	-5.8	-6.4	0.6
RFK	RF	19.5	-6.9	-5.9	-0.9
RFK	FMN	n.d.[a]	n.d.[a]	n.d.[a]	n.d.[a]
RFK:RF	ADP	17.1	-6.5	-31.8	25.4
RFK:RF	ATP	50.4	-5.9	-72.5	66.6
RFK:FMN	ADP	14.8	-6.6	-15.9	9.3
RFK:FMN	ATP	0.5	-8.6	-17.9	9.3
RFK:ATP	FMN	23.9	-6.3	-21.7	15.4
RFK:ATP	RF	n.d.[a]	n.d.[a]	n.d.[a]	n.d.[a]
RFK:ADP	FMN	6.4	-7.1	-17.7	10.6
RFK:ADP	RF	14.5	-6.6	-74.3	67.7

[a] n.d. Not detected. Not heat of interaction was detected for this titration. [b] n.m. Not measured. This combination of ligands in the presence of Mg^{2+} leads to the catalytic reaction, preventing determination of binding heats.
